# Tissue Yields for Epidermal Growth Factor Receptor Analysis in Non-Small Cell Lung Cancer Patients in Trinidad and Tobago

**DOI:** 10.7759/cureus.12531

**Published:** 2021-01-06

**Authors:** Aaron Haralsingh, Mark West

**Affiliations:** 1 Surgery, Eric Williams Medical Sciences Complex, St. Joseph, TTO

**Keywords:** epidermal growth factor receptor gene mutation, tissue yield, lung biopsy, trinidad and tobago, image-guided biopsy, fiberoptic flexible bronchoscopy, lung wedge biopsy, non-small cell lung cancer

## Abstract

Introduction

Patients with unresectable non-small cell lung cancer (NSCLC) may benefit from chemotherapy, tyrosine kinase inhibitor (TKI) therapy, or both. TKI therapy may be administered to the subset of patients who harbor the epidermal growth factor receptor (EGFR) mutation. EGFR mutation testing now plays a vital role in the diagnostic work-up of advanced NSCLC patients to determine which patients are more likely to benefit from TKI therapy. The role of surgery in these patients is mostly limited to obtaining an adequate biopsy for histological, immunohistochemical, and EGFR analysis using the least invasive methods possible. It is thought that larger volume samples, such as those obtained from traditional surgical lung biopsies (SLBs), have better yield than small volume samples, such as those obtained from transthoracic needle lung biopsies (TTNLBs), for EGFR analysis.

Aim

The aim of this was to determine which biopsy procedures provide superior yield for EGFR mutation analysis among primary NSCLC patients at the Eric Williams Medical Sciences Complex (EWMSC) and whether these tissue yields are in keeping with international recommendations.

Methods

This is a retrospective, observational study using patient data obtained from the Lung Malignancy Unit, which is based at the EWMSC. The study population was limited to primary NSCLC patients presenting to the EWMSC from January 2014 to June 2017 whose biopsy samples were sent for EGFR testing. Relevant patient data were entered onto a spreadsheet using Microsoft Excel. Patients were classified as having had either an SLB, bronchial biopsy (BB), TTNLB, or some other biopsy procedure. All samples were sent for histological analysis, followed by immunohistochemistry and finally EGFR testing. All EGFR mutation analysis was performed at a single laboratory in the USA. A minimum of 200 tumor cells or 10% tumor content defined an adequate sample for EGFR mutation analysis. Samples that yielded a positive or negative result were considered adequate samples in this study. The number of adequate and inadequate samples for each procedure group was tabulated and the yield was determined as the percentage of adequate samples obtained for each procedure group.

Results

SLBs had superior yield (95.6%) compared to BBs (88.5%) and TTNLB (85%) in obtaining adequate samples for EGFR analysis.

Conclusion

SLBs demonstrated superior yield in attaining adequate tissue samples for EGFR mutation analysis compared to BBs and TTNLBs.

## Introduction

Lung cancer is the most common cause of cancer death worldwide, accounting for 1.59 million deaths [1]. Non-small cell lung cancer (NSCLC) comprises approximately 85% of lung cancer diagnoses and has three pathological subtypes, namely adenocarcinoma, squamous cell carcinoma, and large cell carcinoma. Around 38.5% of all lung cancers are adenocarcinoma [2,3].

Almost 70% of NSCLC patients present with unresectable disease and may benefit from chemotherapy [4]. In the past, platinum-based chemotherapy served as the mainstay for the treatment of advanced NSCLC. The advent of molecular testing and development of immune therapies has ushered in an era of personalized medicine regarding the treatment of NSCLC. Clinical trials have demonstrated that treatment with tyrosine kinase inhibitors (TKIs) improved progression-free survival in NSCLC patients who harbor epidermal growth factor receptor (EGFR) mutations. EGFR mutation positive patients have been shown to derive more benefit from TKIs than conventional chemotherapy [5,6]. The superiority of TKIs over platinum-based chemotherapy has also been demonstrated among never-smokers of Asian origin with lung adenocarcinoma, even when used as a second-line therapy [7,8]. As such, mutation testing now plays a vital role in the diagnostic work-up of advanced NSCLC patients to determine which patients are more likely to receive benefit from targeted therapies.

In patients presenting with unresectable disease, the goal is to establish a tissue diagnosis using the least invasive methods possible. Minimally invasive procedures confer the benefit of fewer complications, shorter hospital stays, and reduced morbidity for patients [9]. It is thought that larger volume biopsy samples are more adequate than small volume samples in ensuring that adequate tissue is available for EGFR analysis. The biopsy specimens obtained through these procedures are often the only tissue samples available for histological confirmation of cancer, immunohistochemistry (IHC) testing, and mutational analysis. Ensuring the adequacy of the sample for mutational analysis is therefore essential to patient outcomes. Both surgeons and physicians need to be aware of the tissue yields associated with various biopsy procedures.

The Eric Williams Medical Sciences Complex (EWMSC) is the sole referral center for thoracic surgery and medicine in the public healthcare system of Trinidad and Tobago. All patients who access the public healthcare system and are suspected of having lung cancer are evaluated at the EWMSC. These patients are then followed up by the Lung Malignancy Unit (LMU), which promotes lung cancer awareness, facilitates the lung cancer multi-disciplinary team meeting, co-ordinates diagnostic services for patients, and collects data regarding all lung cancer patients within the public healthcare system of Trinidad and Tobago. At our institution, a tissue diagnosis of lung cancer is most often sought via thoracoscopy, mediastinoscopy, bronchoscopy, thoracotomy or transthoracic needle lung biopsy (TTNLBs). In this study, we grouped the various biopsy procedures as follows: surgical lung biopsies (SLBs), bronchial biopsies (BBs), TTNLBs, and others, which included various miscellaneous procedures such as core biopsies of chest wall masses. Image-guided TTNLB procedures have been performed by our interventional radiologists since 2014 and have been sought after with greater frequency over the past years. The tissue yields of these procedures for EGFR testing have not been studied or documented in Trinidad and Tobago, nor within the English-speaking Caribbean.

## Materials and methods

Data acquisition

This is a descriptive, retrospective study carried out at a tertiary healthcare facility (the EWMSC) in Trinidad and Tobago utilizing patient data obtained from the LMU, which is based at the EWMSC and is under the directive of the North Central Regional Health Authority (NCRHA) of Trinidad and Tobago. Permission for data acquisition was obtained from the Chairman of the LMU and ethical approval from the Ethics Committee of the NCRHA.

Study population

The study population was chosen among patients presenting to the EWMSC from January 2014 to June 2017. SLBs and BBs have been performed at our institution for many years, but TTNLB services commenced in 2014; therefore, patients presenting before this time were excluded from this study. Only patients with primary NSCLC whose biopsy samples were sent for EGFR testing were included.

Biopsy procedures

Any wedge resection, lobectomy, pneumonectomy, or any other procedure where tissue samples of the lung were obtained and was performed via an open thoracotomy, video-assisted thoracoscopy (VATS), or anterior mediastinotomy or mediastinoscopy (Chamberlain procedure) was considered an SLB. Tissue samples taken via a flexible or rigid bronchoscope were considered BBs. Bronchoalveolar lavage and brush samples that were sent for cytology and subsequently EGFR testing were not included in this study as they were not considered to be biopsy samples. Any CT- or ultrasound-guided needle lung biopsy was considered a TTNLB. TTNLBs were performed using a co-axial needle system where a minimum of three passes were made with the biopsy needle. Other biopsy procedures included incisional core biopsies of chest wall masses and excisional lymph node biopsies.

Tissue analysis

All samples were sent for histological analysis followed by IHC and finally EGFR testing. Formalin-fixed, paraffin-embedded biopsy samples were sent for EGFR testing at the Cancer Genetics Incorporated Laboratories, Rutherford, New Jersey, United States, where an assay using polymerase chain reaction, pyrosequencing, and Qiagen EGFR Plug-in Report was used to detect EGFR mutations. A minimum of 200 tumor cells or 10% tumor content defined an adequate sample for EGFR mutation analysis. Samples that yielded a positive or negative result were considered adequate samples in this study.

Data aalysis

Patients’ demographic data, smoking status, stage, biopsy procedure, tumor histology, and EGFR status were entered onto a spreadsheet using Microsoft Excel. The number of adequate and inadequate samples for each procedure group was tabulated, and the yield was determined as the percentage of adequate samples obtained for each procedure group.

## Results

From January 2014 to June 2017, 143 patients had biopsy samples tested for EGFR mutation. A total of 138 were classified as primary NSCLC; of these, 130 (94%) were classified as cases of primary lung adenocarcinoma. After applying the inclusion and exclusion criteria and due to limitations in accessing patient records, the data for 103 patients with primary NSCLC were analyzed.

Of the 143 patients, 72 (69.9%) were male and 31 (30.1%) were female. The mean age of presentation of these patients was approximately 63 years. The eldest and youngest patients were 83 and 35 years old at presentation, respectively. The majority reported a smoking history (61.2%); 93.7% (n=59) of those with a smoking history were male, whereas 67.5% (n = 27) of never-smokers were female. The mean age of presentation of both smokers and never-smokers was similar. A total of 95 (92.2%)patients had adenocarcinoma, four had squamous cell carcinoma, two had large cell carcinoma, and one had mixed histology, whereas one patient had NSCLC, not otherwise specified. These data are summarized in Table 1.

**Table 1:** Summary of study data EGFR, epidermal growth factor receptor; SLB, surgical lung biopsy; BB, bronchial biopsy; TTNLB, transthoracic needle lung biopsy

	All patients	EGFR-positive patients	EGFR-negative patients	Insufficient tissue
Sex				
Male	72 (69.9%)	18 (48.6%)	50 (86.2%)	4 (44.4%)
Female	31 (30.1%)	19 (51.4%)	8 (13.8%)	5 (55.6%)
Age				
Mean	62.6	61.8	63.5	60.6
Maximum	83	79	83	81
Minimum	35	40	35	47
Smoking status				
Smoking history	63 (61.2%)	12 (32.4%)	46 (80.7%)	5 (55.6%)
Never-smoker	40 (38.8%)	25 (67.6%)	11 (19.3%)	4 (44.4%)
Biopsy procedure				
SLB	46 (44.7%)	17 (45.9%)	27 (47.4%)	2 (22.2%)
BB	27 (26.2%)	11 (29.7%)	13 (22.8%)	3 (33.3%)
TTNLB	20 (19.4%)	6 (16.2%)	11 (19.3%)	3 (33.3%)
Other	10 (9.7%)	3 (8.1%)	6 (10.5%)	1 (11.1%)

**Figure 1:**
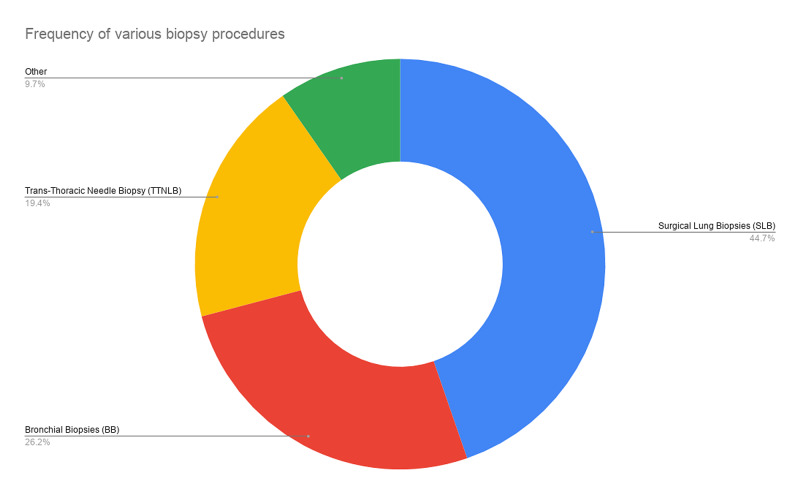
Frequency of biopsy procedures

EGFR analysis was performed on tissue samples obtained through SLB (44.7%), BB (26.2%), TTNLB (19.4%), and other methods (9.7%) (Figure 1).

**Figure 2:**
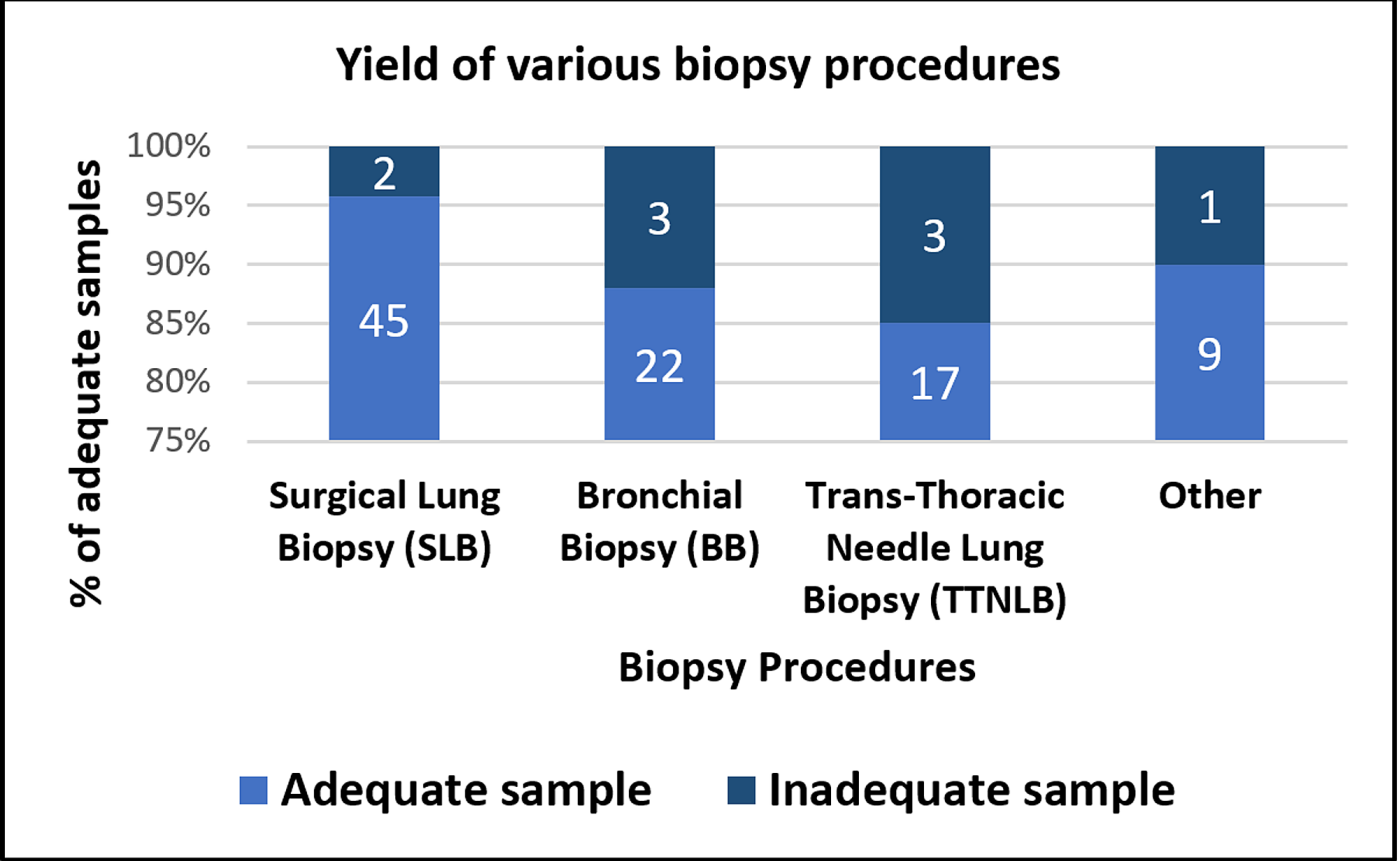
Diagnostic yield of various biopsy specimens

For all biopsy procedures, 94 (91.2%) of 103 yielded adequate tissue for EGFR testing. Of the nine samples deemed insufficient for EGFR analysis, three of them were obtained through TTNLB, three through BB, two through SLB, and one from a cervical lymph node biopsy. The EGFR mutation rate was higher among BB specimens (40.7%) compared to SLB specimens (37%); 30% of all other biopsy specimens (TTNLB specimens and others) were EGFR positive. SLB had superior yield (95.6%) compared to BB (88.5%) and TTNLB (85%) in obtaining adequate samples for EGFR analysis (Figure 2).

## Discussion

Most patients with NSCLC present with unresectable disease; therefore, the acquisition of an adequate biopsy specimen for histological analysis, IHC, and EGFR testing is important in determining the appropriate management of their disease. Studies comparing the tissue yields of various procedures for EGFR testing are sparse and have not been previously undertaken in Trinidad and Tobago. Lung wedge resection specimens are thought to be the “gold standard” for the diagnosis of lung cancer [10]. The adequacy of SLB and BB specimens for EGFR testing is well known, and recent studies have demonstrated that TTNLB is also a safe and effective method of obtaining tissue samples for molecular analysis [11,12]. TTNLB and EGFR testing services have only recently become available in our healthcare system. The tissue yields for EGFR testing associated with various procedures should be known to both surgeons and physicians alike, such that the best decisions can be taken.

In concordance with international data [1,2], male smokers accounted for most of the NSCLC study population, and adenocarcinoma was found to be the most common histological subtype. With a yield of 95.6%, SLBs were found to be superior to BBs, TTNLBs, and other methods for obtaining adequate samples for EGFR analysis at our center. Our results suggest that SLB procedures (such as VATS lung biopsy) should be the procedure of choice for obtaining tissue samples for diagnosis, subtyping, and EGFR analysis in patients with a lung lesion in whom lung cancer is suspected. These results support the notion that larger volume samples are more adequate for molecular testing than small volume samples. Performing biopsy procedures with the best yield in the first instance may reduce the likelihood of repeat procedures, hospital re-admissions, and total length of hospital stay, thus saving time and reducing cost while minimizing patient anxiety in the rough road to lung cancer diagnosis and treatment.

Some European authors recommend that BB yield should be at least 85% when an endobronchial lesion is visible and that radiology-guided percutaneous biopsy yield should be at least 90% for lesions with a diameter of greater than 15 mm [13]. At our institution, patients with a peripheral lung lesion of at least 10 mm in diameter may undergo a TTNLB. As a result, some patients in our study population whose tissue samples were obtained through TTNLB may have had lesions with a diameter of less than 15 mm, which may account for the slightly lower yield (85%) in our study compared to the recommendations. Various studies have demonstrated yields of 70-90% for bronchoscopy and 80-95% for transthoracic needle biopsies [14]. The tissue yields of BB (88.5%) and TTNLB (85%) procedures in our setting are therefore acceptable for EGFR analysis and as such should be considered as viable alternatives where an SLB is undesirable. Based on our results, we would recommend BBs for patients with central lung lesions and TTNLBs for patients with peripheral lung lesions as alternatives to surgical biopsy procedures.

Limitations of this study included its retrospective design, small sample, and limited data retrieval. The length of time elapsed between retrieval of the specimen to its preparation and testing would have varied for each patient, and it is possible that the quality of the samples may have degraded over time. However, timeline data were inadequate to allow for this analysis. This may potentially be a confounding variable in our study. All procedures are operator-dependent, and no data were available to determine whether more experienced operators yielded better tissue samples for analysis. Although this is a single-center study, the EWMSC is the sole thoracic surgical and medical service provider in Trinidad and Tobago, thus providing a sample of the total NSCLC population. Further research into the rate of EGFR mutations, the outcomes of various treatment methods, and the safety and complication rates of various biopsy procedures among our lung cancer population, as well as effects of time and experience of the operator on the quality of specimens obtained for molecular analysis is much needed in Trinidad and Tobago. This knowledge would pave the way for better decision-making in lung cancer diagnosis.

## Conclusions

The tissue yields for EGFR mutation analysis associated with various biopsy procedures in NSCLC was previously undocumented in Trinidad and Tobago. SLBs provide superior samples for EGFR mutation testing compared to other techniques. The tissue yields of BBs and TTNLBs for EGFR mutation testing in our study population are acceptable and can be offered as alternatives to surgical lung biopsies to patients who are unfit for or refuse surgery.
